# Leucine-Rich Repeat Kinase 2 (LRRK2) in Glucose Metabolism and Metabolic–Neuroinflammatory Crosstalk

**DOI:** 10.3390/biom16040588

**Published:** 2026-04-15

**Authors:** Fumitaka Kawakami, Motoki Imai, Masanori Ogata, Toshiya Habata, Shun Tamaki, Rei Kawashima, Yoshifumi Kurosaki, Sayaka Miyai, Moragot Chatatikun, May Pyone Kyaw, Kenichi Ohba

**Affiliations:** 1 Department of Regulation Biochemistry, Graduate School of Medical Sciences, Kitasato University, 1-15-1, Kitasato, Minami-ku, Sagamihara 252-0373, Kanagawa, Japan; 2 Department of Health Administration, School of Allied Health Sciences, Kitasato University, Sagamihara 252-0373, Kanagawa, Japan; 3 Regenerative Medicine and Cell Design Research Facility, School of Allied Health Sciences, Kitasato University, Sagamihara 252-0373, Kanagawa, Japan; 4 Department of Molecular Diagnostics, School of Allied Health Sciences, Kitasato University, Sagamihara 252-0373, Kanagawa, Japan; 5 Department of Applied Tumor Pathology, Graduate School of Medical Sciences, Kitasato University, Sagamihara 252-0373, Kanagawa, Japan; 6 Department of Physiology, School of Allied Health Sciences, Kitasato University, Sagamihara 252-0373, Kanagawa, Japan; 7 Department of Brain Science, Graduate School of Medical Sciences, Kitasato University, Sagamihara 252-0373, Kanagawa, Japan; 8 Department of Occupational Therapy, School of Allied Health Sciences, Kitasato University, Sagamihara 252-0373, Kanagawa, Japan; 9 Department of Medical Laboratory Sciences, School of Allied Health Sciences, Kitasato University, Sagamihara 252-0373, Kanagawa, Japan; 10 Department of Medical Laboratory Sciences, School of Health Sciences, Kitasato University, Minamiuonuma 949-7241, Niigata, Japan; 11 Department of Medical Technology, School of Allied Health Sciences, Walailak University, Nakhon Si Thammarat 80160, Thailand; 12 Research Excellence Center for Innovation and Health Products (RECIHP), Walailak University, Nakhon Si Thammarat 80160, Thailand; 13 Department of Biochemistry, University of Medicine 1, Pyae Road, Kamayut Township 11041, Yangon, Myanmar

**Keywords:** LRRK2, insulin signaling, glucose metabolism, Rab GTPase, mitochondrial function, inflammation, Parkinson’s disease

## Abstract

Leucine-rich repeat kinase 2 (LRRK2) is a multidomain serine/threonine kinase and a major genetic contributor to Parkinson’s disease (PD). Although LRRK2 has been extensively studied in neurodegeneration, emerging evidence indicates that it also plays a critical role in systemic metabolism. LRRK2 regulates glucose homeostasis through modulation of insulin signaling, vesicle trafficking, mitochondrial function, and inflammatory responses. Studies using LRRK2 knockout and knock-in models, including the pathogenic G2019S mutation, have revealed abnormalities in insulin sensitivity, adipose tissue inflammation, hepatic glucose production, and skeletal muscle metabolism. Mechanistically, LRRK2 phosphorylates Rab GTPases, thereby controlling insulin receptor trafficking and GLUT4 translocation. In addition, LRRK2 influences mitochondrial dynamics and reactive oxygen species production, linking metabolic stress to inflammatory signaling. Importantly, LRRK2 also regulates innate immune pathways, including TLR4–NFκB signaling and inflammasome activation, thereby connecting peripheral metabolic dysfunction to neuroinflammation. Here, we propose an integrated metabolic–neuroinflammatory crosstalk model in which LRRK2 functions as a molecular coordinator linking peripheral metabolic dysfunction to central neurodegeneration. In this framework, systemic metabolic stress—characterized by insulin resistance, chronic inflammation, advanced glycation end product (AGE) accumulation, and blood–brain barrier disruption—drives microglial activation and neurodegenerative processes. Understanding this systemic axis may provide new therapeutic opportunities targeting both metabolic dysfunction and neurodegeneration in PD.

## 1. Introduction

Parkinson’s disease (PD) is a progressive neurodegenerative disorder characterized by the selective loss of dopaminergic neurons in the substantia nigra pars compacta and the accumulation of α-synuclein-containing Lewy bodies. While PD has traditionally been considered a brain-specific disorder, increasing evidence suggests that it involves systemic alterations affecting multiple peripheral organs and biological processes [1].

Epidemiological and clinical studies have demonstrated a strong association between PD and metabolic disorders, including insulin resistance, type 2 diabetes mellitus (T2DM), dyslipidemia, and obesity [2,3,4]. Longitudinal analyses further indicate that metabolic abnormalities such as impaired glucose tolerance and insulin resistance may precede the onset of motor symptoms, suggesting a potential contributory role in disease initiation and progression. Mechanistically, metabolic stress can exacerbate oxidative damage, mitochondrial dysfunction, and neuroinflammation [5]—key pathological processes implicated in PD.

Despite these observations, the molecular mechanisms linking peripheral metabolic dysfunction to central neurodegeneration remain incompletely understood. Identifying factors that integrate metabolic and neuroinflammatory pathways is therefore essential for understanding PD pathogenesis.

Mutations in the leucine-rich repeat kinase 2 (LRRK2) gene represent the most common genetic cause of familial PD and are also detected in sporadic cases [6,7,8,9]. Pathogenic variants are distributed across multiple domains of the protein, including R1441C/G/H in the Ras of complex proteins (ROC) domain, Y1699C in the C-terminal of ROC (COR) domain, and I2020T and G2019S in the kinase domain, suggesting diverse mechanisms of LRRK2 dysregulation. The G2019S substitution confers increased kinase activity and has been widely studied; however, it represents only one aspect of LRRK2-associated pathology [10,11,12].

LRRK2 is widely expressed in both central and peripheral tissues, including neurons, microglia, adipocytes, hepatocytes, skeletal muscle, and circulating immune cells [13,14]. Accumulating evidence suggests that LRRK2 regulates multiple cellular processes, including vesicle trafficking, endolysosomal function, mitochondrial dynamics, autophagy, and innate immune signaling [15,16,17,18,19]. These processes are closely associated with metabolic regulation and inflammatory responses.

Taken together, these observations support a model in which LRRK2 may function as a molecular integrator linking peripheral metabolic homeostasis with neuroinflammatory and neurodegenerative processes. In this review, we summarize current knowledge on the role of LRRK2 in glucose metabolism and discuss how metabolic dysfunction may contribute to PD through LRRK2-mediated pathways.

## 2. Structure and Biochemical Functions of LRRK2

**Figure 1:**
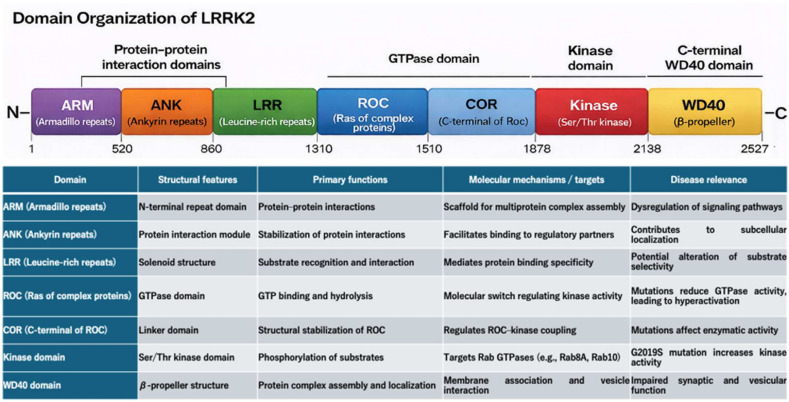
Domain organization and functions of LRRK2. Schematic representation of LRRK2 domain architecture. Each domain contributes to distinct functions, including protein interactions (ARM/ANK/LRR), GTPase regulation (ROC–COR), kinase activity (kinase domain), and protein complex assembly and localization (WD40).

LRRK2 is a ~286 kDa multidomain protein composed of an N-terminal armadillo/ankyrin-like region, leucine-rich repeats (LRR), ROC GTPase domain, COR domain, a serine/threonine kinase domain, and C-terminal WD40 repeats [6,10] (Figure 1). The ROC–COR tandem functions as a GTP-binding module that regulates kinase activity through intramolecular conformational changes. The kinase domain catalyzes phosphorylation of downstream substrates, and the WD40 repeats mediate protein–protein interactions and subcellular targeting. Pathogenic mutations are distributed across multiple domains, including R1441C/G/H in the ROC domain, Y1699C in the COR domain, and I2020T and G2019S in the kinase domain, indicating that distinct molecular mechanisms contribute to LRRK2-associated disease.

Pathogenic variants such as G2019S increase kinase activity and promote hyperphosphorylation of substrates [10,11]. While G2019S is the most extensively studied mutation, other variants also alter GTPase activity, protein interactions, and subcellular localization, highlighting the complexity of LRRK2 regulation. Phosphoproteomic analyses identified multiple Rab GTPases—including Rab8A, Rab10, Rab12, and Rab29—as direct LRRK2 substrates [13,20,21]. Rab phosphorylation at conserved threonine/serine residues alters effector binding and subcellular localization, thereby modulating vesicle trafficking, endocytosis, and lysosomal dynamics [13,20]. These findings suggest that LRRK2-mediated Rab phosphorylation may influence vesicle trafficking and intracellular signaling, although the extent to which this mechanism operates in vivo remains under investigation.

These trafficking pathways are integral to metabolic control. Insulin receptor (IR) internalization, recycling, and degradation depend on endosomal sorting, while insulin-stimulated GLUT4 translocation in adipocytes and myocytes requires Rab-dependent vesicular transport. LRRK2-mediated phosphorylation of Rab10 and Rab8A has been implicated in regulating GLUT4 vesicle docking and fusion, providing a potential mechanistic link between LRRK2 kinase activity and glucose uptake [13,17,20]. However, most of these findings are derived from cellular or animal models, and direct evidence in human tissues is limited. In addition, LRRK2 interacts with components of the retromer complex, clathrin adaptors, and cytoskeletal regulators, further influencing receptor trafficking and signal propagation.

Beyond vesicle trafficking, LRRK2 interfaces with the autophagy–lysosome system. LRRK2 has been reported to modulate lysosomal function and autophagy, although results may vary depending on experimental context. It modulates lysosomal positioning, acidification, and substrate clearance, partly through Rab7/9 pathways and mTORC1 signaling [18,19]. LRRK2 is also associated with mitochondrial membranes and cytoskeletal elements, linking it to organelle dynamics and intracellular transport. Collectively, these observations position LRRK2 as a multifunctional regulator, although its precise physiological roles remain incompletely defined [15,16,22].

## 3. LRRK2 in Inflammation and Immunometabolism

LRRK2 is highly expressed in innate immune cells, including macrophages and microglia, and regulates multiple inflammatory pathways [22,23].

### 3.1. TLR4–NFκB Signaling

LRRK2 has been reported to enhance Toll-like receptor signaling, particularly TLR4, leading to activation of NFκB and transcription of pro-inflammatory cytokines such as TNF-α, IL-6, and IL-1β [22]. LRRK2 is involved in regulating TLR4–NFκB signaling; however, its effects appear to be context- and cell-type dependent. Chronic activation of these pathways contributes to systemic insulin resistance by interfering with IRS signaling and promoting serine phosphorylation of key metabolic intermediates [24]. This association is supported by experimental studies, although the extent to which LRRK2 directly drives these processes in humans remains unclear. In the central nervous system, similar signaling in microglia drives neuroinflammation [25]. LRRK2 may amplify microglial responses to inflammatory stimuli, thereby contributing to neuroinflammatory processes.

In addition to TLR4–NFκB signaling, LRRK2 appears to be regulated by a broader network of inflammatory pathways integrating cytokine signaling, nucleic acid sensing, mitochondrial stress, and lysosomal dysfunction. Notably, interferon gamma (IFN-γ) has been shown to induce LRRK2 expression in immune cells, including macrophages and dendritic cells [26], indicating that LRRK2 participates in cytokine-driven inflammatory programs and may influence the magnitude and persistence of inflammatory responses. Furthermore, cytosolic DNA sensing through the STING pathway, which can be activated by mitochondrial DNA released during mitochondrial stress, may intersect with LRRK2-dependent inflammatory regulation [27,28]. Given that mitochondrial dysfunction and impaired autophagy–lysosome pathways are central features of Parkinson’s disease, these pathways may converge on LRRK2 to amplify inflammatory signaling and immunometabolic dysfunction. In this framework, LRRK2 may function as a convergent regulator linking cytokine signaling, innate immune sensing, and organelle stress, thereby contributing to both peripheral metabolic abnormalities and central neuroinflammatory processes.

### 3.2. NLRP3 Inflammasome Activation

LRRK2 has been implicated in activation of the NLRP3 inflammasome, which processes pro–IL-1β and pro–IL-18 into their mature forms [29,30]. This relationship is supported by experimental evidence, although the precise molecular mechanisms remain to be fully defined. Inflammasome activation can be triggered by mitochondrial ROS, lysosomal damage, and metabolic stress [31]—processes may be influenced by LRRK2 activity. These observations suggest a potential link between LRRK2, mitochondrial dysfunction, and inflammatory signaling. This may create a feed-forward loop linking metabolic dysfunction, mitochondrial damage, and inflammation. However, the strength and directionality of this relationship may vary depending on the experimental context.

### 3.3. Crosstalk Between Metabolism and Immunity

Metabolic states influence immune cell function and vice versa [32]. LRRK2 integrates these processes by regulating both metabolic pathways (e.g., mitochondrial function) and immune signaling. In adipose tissue, LRRK2-driven macrophage activation may promote insulin resistance; in the brain, microglial activation may contribute to synaptic dysfunction and neuronal loss. Therefore, LRRK2 may act as a regulator of immunometabolism rather than a single upstream driver. Thus, LRRK2 sits at the nexus of immunometabolism. However, its relative contribution compared with other regulatory pathways remains to be determined.

### 3.4. LRRK2 in Inflammatory Bowel Disease and Mesenteric Immunometabolism

LRRK2 has been genetically associated with Crohn’s disease and inflammatory bowel disease [33], suggesting a broader role in immune regulation beyond the central nervous system. In IBD, mesenteric adipose tissue functions as an immunometabolic organ, producing inflammatory cytokines and adipokines that influence intestinal inflammation and barrier integrity. LRRK2 may also be expressed in adipocytes and immune cells within mesenteric adipose tissue, where it could be involved in regulating inflammatory signaling, mitochondrial function, and cellular metabolism. Dysregulation of these processes may contribute to intestinal inflammation, increased permeability, and systemic metabolic alterations, including insulin resistance.

Furthermore, emerging evidence suggests that intestinal inflammation may influence central nervous system pathology via the gut–brain axis [34]. In this context, LRRK2 may function as a molecular link between intestinal inflammation and neuroinflammatory processes, potentially contributing to the initiation and progression of PD.

Notably, this integrative role positions LRRK2 as a potential therapeutic target across both metabolic and inflammatory diseases. Targeting LRRK2-dependent pathways may therefore represent a strategy to simultaneously modulate peripheral inflammation and central neurodegenerative processes.

## 4. LRRK2 in Peripheral Glucose Metabolism

### 4.1. Adipose Tissue

Adipose tissue is a major determinant of systemic insulin sensitivity through glucose uptake, lipid buffering, and adipokine secretion. LRRK2 is expressed in both mature adipocytes and stromal vascular cells, including macrophages [14,35]. During adipogenesis, LRRK2 influences transcriptional programs governing lipid storage and insulin responsiveness. Loss-of-function models show altered adipocyte differentiation and lipid handling, whereas kinase-hyperactive states promote inflammatory activation of adipose macrophages. Importantly, Lrrk2-knockout mice fed a high-fat diet e have been reported to exhibit improved glucose tolerance and insulin sensitivity compared with wild-type controls, although these findings are derived from animal models and require further validation in human studies [35].

Mechanistically, LRRK2 has been reported to enhance pro-inflammatory cytokine production (TNF-α, IL-6, MCP-1) via TLR (Toll-like receptor)–NFκB signaling [23,24,26], creating a chronic low-grade inflammatory milieu that interferes with insulin receptor substrate (IRS) function through serine phosphorylation. This impairs downstream PI3K–AKT signaling [36] and may reduce GLUT4 translocation, thereby potentially decreasing insulin-stimulated glucose uptake. In parallel, Rab10-dependent trafficking of GLUT4 vesicles is directly regulated by LRRK2, linking kinase activity to membrane insertion of glucose transporters [13,17,35]. However, this mechanism has been primarily demonstrated in cellular and animal models, and its relevance to human physiology remains to be established. LRRK2 may also modulate adipokine profiles (e.g., adiponectin, leptin), thereby influencing systemic insulin sensitivity.

### 4.2. Liver

The liver maintains glucose homeostasis by balancing glycogen storage and gluconeogenesis. Insulin suppresses hepatic glucose production via PI3K–AKT signaling, which inhibits FOXO1-driven transcription of gluconeogenic genes such as phosphoenolpyruvate carboxykinase (PEPCK) and glucose-6-phosphatase (G6Pase). LRRK2 activity has been suggested to intersect with this pathway, potentially influencing AKT phosphorylation and hepatic glucose output, although the underlying mechanisms remain incompletely understood.

LRRK2 may also influence hepatic oxidative stress and mitochondrial function [16,37,38]. Elevated ROS and impaired oxidative phosphorylation are associated with insulin resistance [38,39]; however, whether LRRK2 directly drives these changes or acts indirectly through inflammatory signaling remains unclear. In addition, LRRK2-mediated alterations in autophagy–lysosome function may affect lipid droplet turnover and lipophagy, contributing to hepatic steatosis and metabolic inflexibility [40].

### 4.3. Skeletal Muscle

Skeletal muscle accounts for the majority of insulin-stimulated glucose disposal. LRRK2 is expressed in myofibers and has been linked to mitochondrial respiration, fatty acid oxidation, and ROS balance [37]. Dysregulated LRRK2 activity has been associated with impaired mitochondrial oxidative capacity in experimental models, which may reduce insulin-stimulated glucose uptake. LRRK2 may also regulate GLUT4 vesicle trafficking in myocytes via Rab phosphorylation, paralleling mechanisms described in adipocytes [13,17,35]. Although fewer studies have addressed muscle-specific roles, available evidence supports a contributory role of LRRK2 in whole-body glucose homeostasis through coordinated effects across adipose tissue, liver, and muscle. However, studies addressing muscle-specific roles of LRRK2 remain limited.

## 5. Molecular Mechanisms Linking LRRK2 to Insulin Signaling

### 5.1. PI3K–AKT–GSK3β Axis

Insulin binding to the insulin receptor activates IRS proteins and PI3K, leading to AKT phosphorylation and activation [41]. AKT phosphorylates GSK3β, inhibiting its activity and promoting glycogen synthesis [42]. LRRK2 has been reported to modulate this pathway at multiple levels; however, whether these effects are direct or mediated through secondary mechanisms such as inflammatory signaling remains under investigation. Enhanced LRRK2 kinase activity has been associated with reduced AKT phosphorylation, either through impaired receptor trafficking or increased inflammatory signaling [43]. This observation is consistent with impaired insulin signaling, although causality has not been fully established. Consequent disinhibition of GSK3β can impair glycogen synthesis and alter phosphorylation of neuronal substrates such as tau, providing a molecular bridge between metabolic dysregulation and neurodegeneration. In line with this, LRRK2 has been reported to regulate tau phosphorylation via GSK3β-dependent mechanisms [44]. However, the extent to which this mechanism contributes to disease progression in vivo remains unclear.

### 5.2. mTOR Signaling, Autophagy, and Nutrient Sensing

mTORC1 integrates nutrient, energy, and growth factor signals to regulate protein synthesis and autophagy. LRRK2 interacts with mTOR pathway components and lysosomal machinery, influencing mTORC1 localization and activity [18,19]; however, its role in autophagy regulation is not fully explained by mTOR-dependent mechanisms alone, as previous studies have demonstrated that LRRK2 can also regulate macroautophagy independently of mTOR and ULK1 signaling [18]. In particular, pharmacological inhibition or genetic suppression of LRRK2 has been shown to induce autophagy through a Beclin-1–dependent mechanism that does not require canonical mTORC1 inhibition [18], suggesting that LRRK2 may directly influence autophagy initiation or vesicle nucleation processes through modulation of Beclin-1 complexes or associated regulatory proteins. Hyperactive LRRK2 has been reported to disrupt autophagic flux, leading to accumulation of damaged organelles and protein aggregates; however, findings vary depending on experimental systems [18,19]. In metabolic tissues, impaired autophagy can exacerbate insulin resistance by promoting lipid accumulation, ER stress, and inflammatory signaling [40], and in the context of Parkinson’s disease, such mTOR-independent regulation of autophagy is particularly relevant because impaired clearance of damaged mitochondria and aggregation-prone proteins such as α-synuclein represents a key pathological feature [18,45]. These associations are supported primarily by experimental studies, and their relevance to human physiology requires further investigation. Together, these observations indicate that LRRK2-dependent regulation of autophagy involves both mTOR-dependent and mTOR-independent mechanisms, highlighting the complexity of its role in cellular homeostasis and disease.

### 5.3. Rab-Mediated Receptor Trafficking and GLUT4 Translocation

Rab GTPases orchestrates the trafficking of insulin receptors and GLUT4-containing vesicles [46]. LRRK2-dependent phosphorylation of Rab8A and Rab10 alters their interaction with effectors required for vesicle docking and fusion [13,20]. These findings suggest that LRRK2 may influence GLUT4 surface expression and glucose uptake; however, most evidence is derived from in vitro or animal studies. In adipocytes and myocytes, this can reduce GLUT4 surface expression and glucose uptake [17,35]. Conversely, genetic deletion or pharmacological inhibition of LRRK2 enhances insulin-dependent GLUT4 translocation and glucose uptake in adipocytes [35,47], supporting a potential role for LRRK2 kinase activity in insulin resistance, although further validation in human systems is required. Additionally, altered endosomal sorting of insulin receptors may change receptor recycling versus degradation, modulating signal duration and intensity. These Rab-dependent processes represent a plausible mechanistic link between LRRK2 activity and insulin responsiveness, but the strength of evidence varies across experimental contexts.

## 6. LRRK2 and Mitochondrial Metabolism

Mitochondria are central to cellular energy production and redox homeostasis [48]. LRRK2 regulates mitochondrial dynamics by influencing fission–fusion balance and mitochondrial transport along cytoskeletal tracks [16,37]. The G2019S mutation has been associated with mitochondrial fragmentation, increased reactive oxygen species (ROS) production, and reduced ATP synthesis, although these findings may vary across experimental systems [37,49,50].

LRRK2 also participates in mitophagy, the selective autophagic removal of damaged mitochondria [18,19]. Impaired mitophagy has been observed in some experimental models of LRRK2 dysfunction; however, the extent to which this contributes to metabolic dysregulation and neurodegeneration remains to be fully established. Impaired mitophagy may lead to accumulation of dysfunctional mitochondria, amplifying oxidative stress and inflammatory signaling [27,28,45]. While this mechanism is supported by experimental evidence, its relevance to human disease progression requires further investigation. In metabolic tissues, these defects translate into reduced oxidative capacity and insulin resistance [51,52]. In neurons, they increase susceptibility to degeneration [45,53]. These associations suggest a link between mitochondrial dysfunction and both metabolic and neurodegenerative processes, although causality has not been definitively established. Furthermore, LRRK2 may regulate mitochondrial DNA stability and electron transport chain efficiency, linking kinase activity to broader aspects of cellular metabolism [37,49]. However, the precise molecular mechanisms underlying these effects remain incompletely understood.

## 7. Metabolic–Neuroinflammatory Crosstalk

Peripheral metabolic dysfunction can influence central nervous system pathology through humoral and cellular pathways [54]. In particular, adipose tissue inflammation, insulin resistance in skeletal muscle, and hepatic metabolic dysfunction contribute to increased levels of circulating cytokines and lipotoxic metabolites [36]. These circulating factors may promote blood–brain barrier (BBB) dysfunction, altered endothelial signaling, and increased immune cell infiltration, although the extent of these effects likely depends on physiological and pathological context [55,56,57,58]. Notably, advanced glycation end products (AGEs) may directly impair BBB integrity through receptor for AGE (RAGE)-mediated endothelial activation and oxidative stress, thereby facilitating the entry of inflammatory mediators into the central nervous system [57,59]. In addition, LRRK2 expression in vascular and immune cells may modulate endothelial responses and barrier function, suggesting a potential role in regulating BBB integrity under inflammatory conditions [24].

In addition to these factors, AGEs—non-enzymatically formed through reactions between reducing sugars and proteins or lipids and accumulated under conditions of chronic hyperglycemia and metabolic stress—may further amplify systemic and central inflammation [59]. AGEs activate RAGE, leading to oxidative stress and amplification of inflammatory signaling cascades [59]. Notably, neurons expressing the G2019S-LRRK2 mutation exhibit increased susceptibility to AGE-induced cytotoxicity, accompanied by upregulation of RAGE expression, suggesting enhanced AGE–RAGE signaling under pathogenic conditions [60]. These findings indicate that G2019S-LRRK2 may sensitize neuronal cells to AGE-mediated stress, thereby amplifying downstream inflammatory and oxidative responses [60].

Furthermore, insulin resistance in skeletal muscle and liver plays a central role in disrupting systemic metabolic homeostasis and establishing a chronic inflammatory state. Reduced glucose uptake in skeletal muscle and increased hepatic gluconeogenesis contribute to persistent hyperglycemia, which in turn promotes AGE formation. This metabolic environment may interact with adipose tissue-derived inflammatory cytokines to further exacerbate systemic inflammation and oxidative stress.

Within the brain, microglia respond to systemic inflammatory cues and metabolic stress by adopting pro-inflammatory phenotypes [54]. LRRK2 may amplify these responses, leading to increased production of neurotoxic mediators [24]. Although LRRK2 may enhance microglial responsiveness to peripheral inflammatory signals, it likely functions as a modulatory regulator rather than a sole driver of neuroinflammation. These mediators may promote phosphorylation and aggregation of α-synuclein and hyperphosphorylation of tau, impair synaptic function, and accelerate neuronal loss [61,62]. These observations are supported by experimental studies, although causal relationships in human disease remain to be fully established.

**Figure 2:**
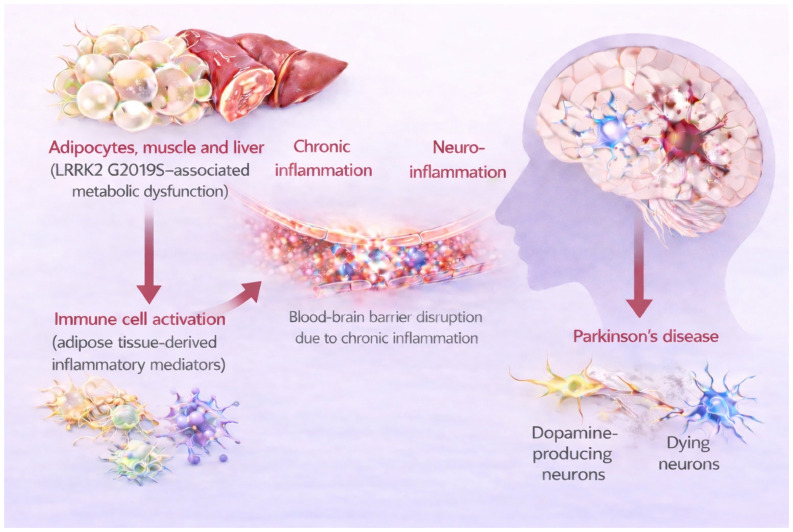
LRRK2-associated metabolic dysfunction promotes systemic inflammation and neurodegeneration leading to Parkinson’s disease. LRRK2-induced abnormalities in metabolic tissues (adipose tissue, skeletal muscle, and liver) enhance the production of inflammatory mediators, driving systemic inflammation and blood–brain barrier disruption. This process facilitates neuroinflammation and dopaminergic neuronal loss, contributing to Parkinson’s disease progression.

This integrated network—linking adipose tissue, skeletal muscle, liver, and brain—provides a conceptual framework for understanding how systemic metabolic disturbances contribute to the pathogenesis and progression of Parkinson’s disease (PD). Within this network, LRRK2 may function as an integrative molecular coordinator rather than a single upstream determinant, translating peripheral metabolic and inflammatory signals into central neuroinflammatory responses [24]. In particular, under conditions characterized by AGE accumulation, insulin resistance, and BBB dysfunction, LRRK2 may mediate the integration of these signals and thereby accelerate neurodegenerative processes. A schematic model illustrating adipose tissue-derived inflammatory mediators, muscle and hepatic metabolic dysfunction, BBB disruption, and subsequent neuroinflammation leading to PD is presented in Figure 2.

## 8. Therapeutic Implications

### 8.1. LRRK2 Kinase Inhibitors

Selective LRRK2 kinase inhibitors (e.g., MLi-2, BIIB122/DNL151) reduce LRRK2 kinase activity and Rab phosphorylation, and can attenuate downstream inflammatory responses [22,63,64]. Preclinical studies in cellular and animal models have demonstrated that these compounds can improve vesicle trafficking, reduce inflammatory signaling, and modulate mitochondrial function [63,65]. Given the role of LRRK2 in metabolic tissues, LRRK2 inhibition may also improve insulin sensitivity and glucose homeostasis [44,47], although this requires further clinical validation. However, clinical evidence remains limited. Early-phase clinical trials have primarily focused on safety, tolerability, and target engagement, and the metabolic effects of LRRK2 inhibition in humans remain to be fully characterized [65].

### 8.2. Metabolic Therapies Targeting LRRK2-Linked Pathways

Interventions that improve metabolic function may indirectly modulate LRRK2 signaling. Insulin sensitizers (e.g., metformin, thiazolidinediones), GLP-1 receptor agonists, and mitochondrial antioxidants have demonstrated neuroprotective and metabolic benefits in experimental models and clinical studies [66,67,68,69]. By reducing systemic inflammation and improving mitochondrial function, these therapies may counteract LRRK2-driven pathological processes. However, whether these effects are mediated directly through LRRK2-dependent pathways or reflect broader improvements in metabolic homeostasis remains unclear. Combination strategies targeting both LRRK2 kinase activity and metabolic dysfunction may therefore offer synergistic therapeutic benefits. Such approaches may be particularly relevant in the context of the metabolic–neuroinflammatory crosstalk, where metabolic, inflammatory, and neurodegenerative processes are interconnected, although this concept requires further experimental validation.

Furthermore, selectively targeting peripheral LRRK2 using brain-impermeable inhibitors may represent a novel preventive strategy. By improving systemic metabolic function and reducing peripheral inflammation without directly affecting central LRRK2 activity, such approaches could modulate metabolic–neuroinflammatory crosstalk and potentially lower the risk of neurodegeneration. However, this concept remains speculative and requires further experimental and clinical validation.

## 9. Future Research Directions

Future research should therefore focus on several key areas. First, tissue-specific functions of LRRK2 in metabolic organs such as adipose tissue, liver, skeletal muscle, and mesenteric adipose tissue need to be more clearly defined. Conditional knockout or knock-in models will be particularly valuable for dissecting how LRRK2 regulates insulin signaling, lipid metabolism, and inflammatory responses in these tissues. Second, the interaction between aging and metabolic stress represents an important but insufficiently explored aspect of LRRK2 biology. Aging is a major risk factor for PD and is closely associated with mitochondrial dysfunction, chronic inflammation, and metabolic dysregulation. Understanding how age-dependent changes in metabolic and immune pathways interact with LRRK2 signaling may provide insight into the temporal progression of disease pathology.

Third, the molecular mechanisms by which Rab-mediated vesicle trafficking regulate receptor signaling and transporter localization in metabolic tissues require further investigation. Given that Rab proteins control endosomal trafficking, receptor recycling, and exocytosis [70], LRRK2-dependent phosphorylation of Rab substrates may broadly influence cellular responsiveness to hormones, cytokines, and metabolic signals. However, the relative contribution of these pathways in different tissues and disease contexts remains unclear. Detailed characterization of these pathways may reveal novel mechanisms linking vesicle trafficking to metabolic disease.

**Figure 3:**
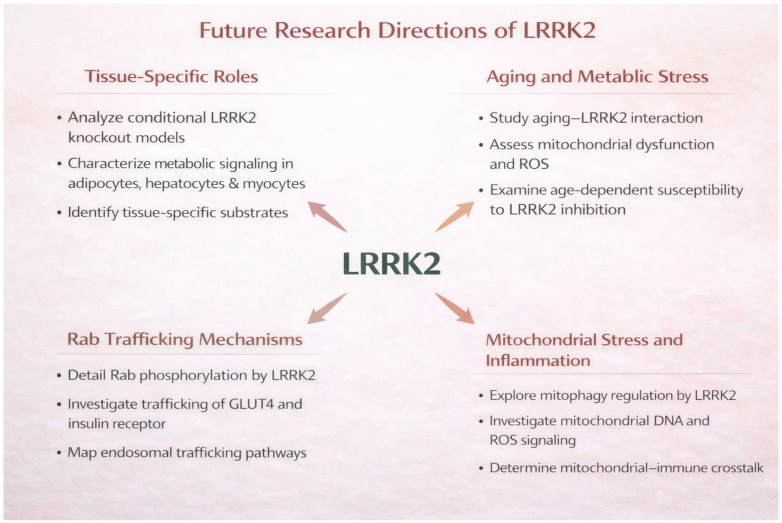
Future research directions for LRRK2 biology in metabolism and neurodegeneration. LRRK2 is emerging as a key regulator linking systemic metabolism, vesicle trafficking, mitochondrial function, and inflammatory signaling. Future studies should clarify (i) tissue-specific roles of LRRK2 in metabolic organs using conditional models; (ii) interactions among aging, mitochondrial dysfunction, ROS, and metabolic stress; (iii) Rab GTPase-dependent regulation of receptor and transporter trafficking, including GLUT4 and the insulin receptor; and (iv) crosstalk between mitochondrial stress, mitophagy, and inflammatory signaling. Elucidating these mechanisms will advance our understanding of the metabolic–neuroinflammatory axis of LRRK2 in PD.

Finally, the integration of mitochondrial quality control pathways with inflammatory signaling represents another promising research direction. In particular, the interplay between mitochondrial dysfunction, reactive oxygen species (ROS), and innate immune activation including inflammasome signaling should be further explored. Mitochondrial dysfunction can activate innate immune responses through the release of mitochondrial DNA, ROS, and other danger-associated molecular patterns. LRRK2 may serve as a regulatory node that coordinates mitochondrial stress responses with immune activation, thereby amplifying neuroinflammatory processes. In addition, future studies should investigate how LRRK2-mediated pathways operate within the framework of metabolic–neuroinflammatory crosstalk, integrating peripheral metabolic dysfunction and central neurodegeneration, as illustrated in Figure 3.

## 10. Limitations and Challenges

Despite accumulating evidence supporting a role for LRRK2 in metabolic regulation, several important limitations should be considered when interpreting current findings.

First, much of the available evidence is derived from in vitro systems or animal models, and the extent to which these observations translate to human physiology remains uncertain. Differences in species, experimental conditions, and genetic backgrounds may significantly influence the observed effects of LRRK2 on metabolic pathways.

Second, LRRK2 exhibits pronounced tissue-specific functions, and its roles may differ substantially across adipose tissue, liver, skeletal muscle, and immune cells. This heterogeneity complicates efforts to define a unified model of LRRK2-mediated metabolic regulation and highlights the need for tissue-specific analyses.

Third, although LRRK2-associated metabolic alterations are increasingly recognized, it remains unclear whether these changes represent a causal driver of Parkinson’s disease pathogenesis or a parallel consequence of systemic dysfunction. Clarifying this relationship will be essential for determining the therapeutic relevance of targeting LRRK2 in metabolic contexts.

Addressing these limitations will be critical for establishing a more comprehensive understanding of LRRK2 as a systemic regulator linking metabolism and neurodegeneration.

## 11. Conclusions

LRRK2 has emerged as a key molecular regulator integrating metabolic regulation, vesicle trafficking, mitochondrial homeostasis, and innate immune signaling. Although originally identified as a genetic cause of familial Parkinson’s disease (PD), accumulating evidence indicates that its roles extend beyond neuronal systems. In particular, LRRK2 contributes to systemic metabolic regulation through kinase-dependent phosphorylation of Rab GTPases, modulation of vesicular trafficking, and regulation of mitochondrial and inflammatory signaling pathways.

Dysregulated LRRK2 activity is increasingly associated with peripheral metabolic abnormalities in addition to neuronal dysfunction. Hyperactivation of LRRK2, particularly in the presence of pathogenic mutations such as G2019S, impairs insulin signaling, reduces glucose uptake, and alters vesicular trafficking of metabolic transporters including GLUT4. These alterations contribute to systemic insulin resistance and metabolic stress. Moreover, LRRK2-mediated phosphorylation of Rab proteins disrupts endosomal and recycling pathways that regulate receptor trafficking, thereby affecting multiple signaling cascades involved in metabolic homeostasis.

Mitochondrial dysfunction represents another key component of LRRK2-associated pathology. LRRK2 regulates mitochondrial dynamics, oxidative phosphorylation, and quality control processes such as mitophagy. Dysregulated LRRK2 activity compromises mitochondrial integrity, leading to increased reactive oxygen species (ROS) production and impaired cellular bioenergetics. These abnormalities enhance neuronal vulnerability while also influencing systemic metabolism in peripheral tissues.

LRRK2 also plays a critical role in innate immune signaling. It enhances Toll-like receptor (TLR)-mediated responses and activates downstream pathways such as NF-κB, resulting in increased production of pro-inflammatory cytokines including TNF-α, IL-6, and IL-1β. Persistent activation of these pathways drives chronic low-grade inflammation, a key contributor to metabolic disorders such as insulin resistance and adipose dysfunction. In addition, peripheral inflammatory signals influence the central nervous system through humoral and cellular mechanisms, including blood–brain barrier disruption and microglial activation.

Collectively, LRRK2-mediated metabolic dysfunction, mitochondrial impairment, and inflammatory signaling form an interconnected network linking peripheral metabolic abnormalities to neurodegeneration in PD. These findings support the concept that Parkinson’s disease is not solely a brain-restricted disorder but a systemic condition involving complex interactions between peripheral metabolism and neuroinflammation. In this framework, metabolically active tissues—including adipose tissue, liver, skeletal muscle, and immune cells—contribute to disease progression through the release of cytokines, metabolic intermediates, and extracellular vesicles that influence brain homeostasis.

Consistent with this systemic model, suppression of LRRK2 activity improves glucose tolerance and metabolic profiles in experimental systems, suggesting that LRRK2 acts as a negative regulator of systemic glucose metabolism. These observations raise the possibility that LRRK2-targeted therapies, currently under development for PD, may also ameliorate metabolic dysfunction. However, given the broad expression of LRRK2 across tissues and its involvement in diverse physiological processes, a detailed understanding of tissue-specific functions will be essential for safe and effective therapeutic targeting.

Overall, LRRK2 occupies a central position at the intersection of metabolic regulation, immune signaling, and neurodegeneration. Elucidating LRRK2-mediated metabolic–neuroinflammatory crosstalk will be critical for understanding PD pathogenesis and may enable the development of therapeutic strategies that simultaneously target metabolic dysfunction and neurodegeneration.

## Data Availability

No new data were created or analyzed in this study.
